# Meeting of the Fulton County Medical Society

**Published:** 1914-09

**Authors:** R. R. Daly


### FULTON COUNTY MEDICAL SOCIETY.

### Regular Meeting September 3, 1914.

By R. R. Daly, M. D.

Dr. S. P. Harris presented a case of a physician who developed symptoms of acute pulmonary tuberculosis in December, 1913. In January Pneumo-thorax treatment was instituted and 10 days later no bacilli were to be found. There were many pleural adhesions, be he was at home three weeks later after the pneumo-thorax and has been well since then. X-Ray plates were made both before and after the disease and these were shown. The patient said that from his point of view as a patient, the treatment was pretty rough but the results were fine and he would quickly repeat the treatment if necessary.

Dr. Willis Jones presented a case of Spontaneous Ennucleation of the Thyroid by Haemorrhage. The boy was 16 years old. He fell from a tree or shed about 12 feet to the ground. This was quickly followed by swelling in the neck and dyspnoea. The next morning he had to hold his head forward in order to breathe. The thyroid appeared to be swollen symmetrically. A diagnosis of haemorrhage in that gland was made. The following morning the breathing was so bad that preparations were made to operate. Cyanosis occurred instantly the anaesthetic was given and quick cuts were made into the tumor to relieve the breathing. Clots came and then two masses were expelled from the opening by slight external pressure. They proved to be the lobes of the thyroid and were exhibited. There was no haemorrhage after the ennucleation. The patient is being watched for myxoedema. There is no case like it recorded so far as Jones knows.

Dr. J. S. Derr exhibited a case of multiple warts on the neck and chin of a man that had been cured by 14 exposures to the x-Ray. There was some erythema, but he expects that to pass and the beard to return.

Dr. E. P. Merritt read a paper on "Epididymotomy," which appears elsewhere in the Journal.

Dr. O. F. Elder said that this operation is just as strongly indicated as that upon pus tubes in women. In fact, the epidymis is what might be well called the "male tube." One should wait only until the swelling is localized. The incision should always be made outside the tunic, otherwise there are adhesions to the testicle and consequent nervous symptoms. Elder thinks it better to give gas than cocaine and then to use a small cigarette drain.

Dr. W. E. Emery said that the paper was interesting and of value to the general practitioner because of the character of the cases that come to every doctor's care. He had himself tried every known means in the care of this disease including ice, poulticing, vaccines, massage, etc., as well as operations and they all result in sterility. Therefore, because it is the simplest and the most successful, epididytomy is the thing to do. Should it be done at the office? Some cases may be so attended, but we should be careful on that point. There is risk of shock and infection. He doesn't approve of Merritt's knife because one can not see what is being done. Better in the long run to dignify the operation and take the patient to the hospital. However, he recognizes that Merritt is a pioneer in this method and is proceeding cautiously. He hopes that the results of the investigations will be gratifying to all.

Dr. E. C. Thrash called attention to the anatomy of the parts and said the tubes were so convoluted that sterility was inevitable if they were cut. No way could be providede for accurate union of the severed parts.

Dr. M. T. Davis said that he has seen several cases of Merritt's and the work has been dextrously and successfully accomplished. His former practice has been to poultice patients in bed every two hours for twenty-four hours, paint the parts with sol nitrate of silver—40 grs. to the ounce and then straps. This gives good results.

E. G. Ballenger said that this operation was a good one. The incision method is quicker and more successful than the dissection.

The convoluted tubules are numerous and thus the pathway from the testes is kept open, but as a matter of fact about 80% of the cases of double epididymitis are sterile. We cut the vas deferens when we want to make a man sterile and this stops all the tubules. For himself, he usually selects gas and four days in the hospital for such patients. Cocaine has not yet been successful in his hands.

Dr. W. L. Champion said that this is a recognized operation for epididymitis and is the best way in most cases. It can be done in 2 or 3 minutes and is as complete as if dissection had been made. It holds the patient because it relieves the pain and does the work with little trouble to him.

Dr. Merritt closed and said that most of the pain in the cocaine method comes from the way the testicle is handled. If this is not grasped too hard, the patient will scarcely ever make a complaint.

Dr. L. C. Fischer said in regard to his Recent Experience in European Hospitals that he had hardly gotten through running well enough to talk. When he was in Rome, there was a strike of some kind and no little violence, so he hastened from there. He was in Vienna when the Servian matter started and he hustled to get out of that city while the going was good. In Germany he encountered considerable fighting and endless crowds of soldiers and many inconveniences, so with all the dispatch he was able to exert, he got away from that country and found himself at The Hague when Liege was besieged. That sort of thing was too close for his comfort and the usual tranquil and meditative state of his mind, so he returned to his home country at the earliest possible moment and with such speed to avoid the laying of mines and the possible encountering of hostile war ships that he really did not breathe leisurely until inside of Sandy Hook and where he could see the railroad station that would give him a good start to Georgia. However, with all this or in spite of it, he found much that was interesting in the European hospitals and a great deal that was novel despite all that he had read about them. Everywhere the immense volume of work and the careful research methods with which it was handled were so impressive that no account of them will do them justice. In Naples they have a museum of comparative instruments and copies of those that were found in Pompeii. A rectal dilator and some gall stone instruments are as good as those we use today; the haemostats were large and cumbersome, but he was surprised to see any at all. The gall stone scoop was just the same. The hospital at Naples was built in 800 A. D. It is made of stone and is very dark and dirty. Apparently the only use they have for water in that locality is to row boats in. At Milan there is a 17th century hospital with a new and fine operating room, which was installed by the King. Venice is old and dirty and degraded and can just be passed over from the surgical point of view. In Rome, the St. Bartholomew's Hospital is on an island in the Tiber and was put there 200 years B. C.

It has been "restored" and has a fairly decent operating room. There is another hospital there, cared for by the government, where Bastinalli does his work and the operating room is the finest in Europe. A great deal of spinal anaesthesia is used there but not with gratifying results from our view point. Fischer said he was shown a piece of rock upon which were the foot prints of Christ as he stood telling Peter he must go to Rome. There were four different sets of these in as many churches and they are venerated to the extent of being used by sick people instead of having proper attention.

At Vienna the first impression of the hospital was that it was the largest livery stable he had ever seen. There were bill boards all over it and the general impression was bad. No bed is free. Insurance is issued to the populace to cover the hospital expense. There are no trained nurses in Vienna. They are just getting round to that necessity and advantage. There are operating nurses, of course, and they do nothing else but work with the one man. Thus the operating technic is marvelous. However, the surgeons do not change their street clothes nor use full gowns—just aprons are fastened on.

Dr. F. W. McRae, speaking on Special Features of British Surgery, said that he has been in London five times since 1901 and there have been splendid changes in the work there since then. At that time, the British surgeon was rather cocky and self satisfied, but the exchange of visits he has made with American surgeons has led to open-mindedness and to mutual adaptation which has improved men on both sides. The British surgeon is first a perfect anatomist and pathologist. He is trained by coming along up through the various grades of hospitals and spends long times in each, working all the time in accord with a definite system as a basis. There results a fine technic with which astonishing things can be accomplished. Echols, of St. Bartholomew's, did a gastro enterostomy without clamps and without hurry, yet he was through in 14 minutes. A bone plating of a fractured femur with plates taken from a phalanx of the finger was completed in 20 minutes and with no sign of haste. Mr. Lane is a marvelous surgeon. He does bone plating entirely with instruments, never allowing any of the parts to be touched with the hands. Cleft palate work is done in babies almost as soon as they are born and with happy results.

Lane's operation removes the entire colon and six inches of the illium and it is done with free manipulation. The intestines were pulled out just as the operator wanted, but it was noticed that there was much salt solution kept running all the time and that the belly was left full of it. This shows that there was much shock and in McRae's opinion it was due to unnecessary manipulation of the intestines.

The hospitals themselves are dirty as compared with ours. McRae stayed some little time after the war was declared but did not have any trouble except with money matters for a few days.

Dr. E. C. Davis spoke of "London Hospitals."

He said that at his first visit things moved so rapidly and in such even sequence that it was like looking at a panorama. What struck him very forcibly is the fact that all the large hospitals are entirely and exclusively for the use of the poor. They are supported by the government. The buildings are old but the operating rooms are modern and complete. The paying patients have to go to private places that are remodelled houses and as a rule are run by some nurses. They are not looked upon with much favor, but they are the only places where paying patients can go. The large hospitals have fine operative systems and organizations, but the nurses do not make a favorable impression. Each hospital is a medical school. A man must remain there at least five years to secure his degree. The surgeons clean up with soap and iodide of mercury 1-5000. The operative fields are cleared with iodine 2%. Plain water is used before the gloves are put on. There seems to be some carelessness, for a man will adjust his head cover after the gloves are on. Silk is used in suturing almost universally. He saw gut used only once, and skin clips only once. The speed of the work without lost motion is wonderful. For instance, he saw the uterus placed upon the abdomen in 2 minutes. Of course there was more to be done, but to get that far in that short time exceeded anything he had ever seen in hysterectomy. In 14 minutes the operation was complete in every detail. There was no abdominal dressing whatever. The part was painted with iodine 2% once a day for several days. The patient is allowed to watch the wound heal. It saves dressings, too.

London was not exercised by the war. One thing amused him greatly. At a surgical congress meeting, a woman suddenly called out, "I am a Mouse, I am just out of jail." She was a suffragette and began to address the meeting, interrupting the speaker. A burly policeman took her under his arm and carried her off. An Englishman remarked that the attitude of all the American doctors present was one of respect because the interruption came from a woman and he said he thought that taught a lesson that might be followed with advantage in handling the excited ladies who make some of the suffragette disturbances.

Mobilization went on in a quiet and dignified way mostly at night when fewer people were disturbed and when almost no one could know what was being done. The variety of uniforms was striking. All the way from that of the Scotch Highlanders to the very gaudy ones of the King's Own Guard.

Dr. H. I. Battey said of "The Orthopedic Work in London," that because of the war he had not had as much time to spend in observation as he wished. He did see some splendid work done and like the other men he was greatly impressed with the team work of the staff and the accurate skill of the operator wherever he went. Notable among the operators were Robert Jones, of Liverpool, and Kellogg, of London. Plaster bandages are not used so much as a sort of loose felt that is saturated with a thick plaster and then moulded to the parts. The surgeons prepare this themselves and take great care with it.

REGULAR MEETING SEPTEMBER 17, 1914.

Dr. F. G. Hodgson presented a case of Hammer Toe. The man weighs 270 pounds and has callouses on the bottoms of the feet and upon the elevated knuckles of the toes. He was greatly crippled thereby. One foot has been operated upon. A resection of one of the metatarsals and of the first phalanges of the foot was done and an arthroplasty made from the tissue taken from the fascia lata. The result was so to change the angle of the metatarsal bones that at the phalangeal joint the pressure would not impinge so much upon the ends of the bones. Thus the hump in the toes themselves was given room in which to subside and the foot became nearly flat at that point. The operation is original and has never been reported before. The after treatment was massage for about a month. The result as shown to the Society was a usable foot with the toes in place and no tenderness remaining. The other foot will be operated upon shortly. The wound in the thigh where the fascia lata was secure to surface the new joints in the foot, healed rapidly.

Dr. Claude A. Smith showed two cases of experimental hook worm infection in negroes. The disease is a tropical one and thus not common among negroes, but the work was taken up to determine, among other things, what effect it might have with the race.

In one case he put sand filled with larvae upon the wrist of a half breed negro. In a short time the itch appeared. In the other case, the man was full bred and the larvae were bound to the wrist in gauze. In one hour 5000 had entered the skin. The rash was like that of poison ivy. It is too early as yet for the worms to have entered the intestines.

Dr. R. R. Kime and Dr. J. S. Derr exhibited a case of gastric ulcer with possible malignancy. The plates Derr showed displayed the projection into the stomach of a small area that is the seat of the difficulty. They also showed 23 hour residue with curvature below the umbilicus.

Dr. C. A. Rhodes exhibited a case of an 8-year-old boy suffering with idiocy of the Mongolian type due to hereditary syphilis.

Dr. G. D. Ayer exhibited a case of a negro girl with an extensive facial burn when a baby. There was cicatrical ectropin. He had done a Woolf graft in three areas and now presents a serviceal lower eyelid.

Dr. Dunbar Roy read a paper on "Acute Mania Following Atropin Drops in the Eye."

Dr. S. A. Vsanski said that the effect seemed to him to be a delirium rather than a mania. It is a good thing to talk of any drug that may give toxic symptoms both for the purpose of making us careful and of restraining its use as far as possible.

Dr. R. R. Daly said that he was glad to note that Roy was using less mydriatics in refraction than formerly. In his own practice, even among children, he rarely found it necessary to use atropin or homatropin. He got better results by measuring an eye when at work than when it was partially paralyzed with mydriatics.

Dr. L. B. Clarke asked if there was any difference in susceptibility to the drug between blondes and brunettes.

Dr. E. C. Cartledge said that with atropin and the symptoms following its toxic administration, there seemed on the surface to be a proof of the principle of similia silibus curantur.

Dr. Roy said that he had not noticed any difference between blondes and brunettes and that his cases rarely went to the toxic extent.

Dr. E. G. Ballenger read a paper on "The Diagnosis of Renal and Bladder Affections." It appears elsewhere in the Journal.

Dr. J. S. Derr said that Dr. Grange, of New Orleans, uses oxygen to dilate the bladder, but air does just as well.

Dr. W. F. Shallenberger said that there have been many cases of mistaken diagnosis in regard to both presence and absence of cystitis, especially among women. The urethra plays an important part in the symptomatology when inflamed. In women the voided urine may show pus without indicating cystitis. There are too many other chances of infection to make this one of use. The sample should always be obtained by catheter. Irrigations improperly used may carry the infection from urethra to the bladder. He spoke of a case where the pains all came from the left side, but repeated ureteral catheterization showed that the diseased kidney was on the right side. It was removed and recovery ensued.

Dr. W. N. Adkins related a case of the removal of a normal kidney through lack of investigation of ureters.

Dr. E. C. Cartledge said that it is a common mistake to overlook pyonephrosis and that the diagnosis of typhoid fever is made instead.

Dr. J. L. Campbell said that he had had a patient with pains on one side, but the x-Ray had shown stone in the other kidney.

Dr. Ballenger in closing urged the repeated examination of many specimens of urine before a final conclusion should be reached.

